# Evaluation of surface electromyographic (SEMG) activity among individuals before and after orthodontic treatment - An observational study

**DOI:** 10.6026/973206300222927

**Published:** 2026-05-31

**Authors:** Manikanda Prabu V, Karthikeyan M.K, Jeevana Poovalingam, Raj Vikram N, Saravana R, Raja Kumar P

**Affiliations:** 1 Department of Orthodontics and Dentofacial Orthopaedics, Thai Moogambigai Dental College and Hospital, Dr. MGR Educational and Research Institute, Chennai 600107, Tamil Nadu, India

**Keywords:** Surface electromyography, orthodontic treatment, temporalis, masseter, digastric, neuromuscular adaptation, malocclusion

## Abstract

Orthodontic treatment initially reduces masticatory muscle activity due to discomfort, but gradual occlusal improvement enhances muscle strength and balance, highlighting the need to assess muscle function before and after treatment. Therefore, it is of interest to evaluate the changes in surface electromyographic (sEMG) activity of masticatory muscles such as temporalis, masseter and digastric muscle before and after orthodontic treatment across different malocclusion. Patients exhibiting malocclusions with class I, II and III were assessed using BioEMG equipment with BioPak analysis software (Digital Dental, NSW and Australia). Temporalis, masseter, and digastric muscles were recorded bilaterally for electromyographic activity at rest, during clenching, and during swallowing. Muscle activity was assessed during orthodontic therapy of pre- and post- treatment values. Shapiro-Wilk test was used to assess the normality of the data and to compare between pre- and post-treatment paired t test was used. During EMG activity across all three muscles, there were no statistically significant difference observed in class I and Class III subjects during pre and post treatment. In contrast, Class II subjects' particularly female subjects showed significant post-treatment increases in EMG activity of the three muscles indicating neuromuscular adaptation to orthodontic treatment. There was a notable increase seen in muscle activity of temporalis, masseter and digastric in Angles Class II females from pre- and post (6 months) of orthodontic treatment.

## Background:

Surface Electromyography (sEMG) for Neuromuscular Assessment Surface electromyography (sEMG) is a non-invasive diagnostic tool that provides objective measurements of muscle function by recording electrical activity during contraction and relaxation [1]. Muscles evaluated using sEMG are four primary muscles assessed in this study include the Temporalis Muscle involved in jaw closure, stabilization and lateral movement. The Masseter Muscle generates occlusal force aiding in mastication. Digastric Muscle generates swallowing and mandibular depression. Orthodontic treatment may lead to neuromuscular adaptation causing changes in these muscle activities [16], which can be objectively quantified using sEMG before and after treatment [1]. Functional neuromuscular adaptation analysis sEMG helps analyze how muscle activity changes post-orthodontic treatment. Previous studies showed that orthodontic and myofunctional treatments can significantly alter sEMG-measured activity in Class II malocclusion patients. The previous study demonstrated that the masseter and temporalis muscles showed a significant increase in Class II division 1 malocclusion patients treated with pre-orthodontic appliance treatment at clenching after 12 months reaching to similar values of untreated normal controls [2]. Similarly, a long term study showed a flexible fixed functional appliance in females revealed a decrease in temporalis and masseter activity during swallowing and maximum clenching that gradually returned to baseline over months which indicates dynamic neuromuscular adaptation [3]. SEMG has been used to assess the association between the morphological parameters such as overjet and muscular activity showed increased overjet can correlate with altered masticatory performance in class II subjects [4]. Despite these findings, there remained a limited observational data in comparing sEMG of muscles such as temporalis, masseter, and digastric across different malocclusion class (Class I, II, III) in both genders before and after fixed orthodontic treatment over a period of six months. Therefore, it is of interest to evaluate masticatory muscle activity (temporalis, masseter & digastric) using sEMG to analyze the neuromuscular adaptation by assessing pre- and post-treatment changes.

## Materials and Methods:

The present study was conducted in Department of Orthodontics and Dentofacial Orthopaedics, Thai Moogambigai Dental College and Hospital, Chennai, India. This prospective *in vivo* study was approved by the Institutional Ethical Clearance Committee (232/2025/IEC/TMDCH). The following criteria were used to select thirty patients undergoing orthodontic treatment.

## Inclusion criteria:

[1] Subjects aged 18-24 years.

[2] Subjects were treated with fixed pre adjusted edgewise appliances (MBT 022 SLOT).

[3] Class I, Class II & Class III orthodontic malocclusion prior to treatment.

[4] Good periodontal & dental health.

## Exclusion criteria:

[1] Medically compromised.

[2] Symptoms indicating temporomandibular disorders (TMD).

## Study setting and equipment:

Measurement of muscle activity using Surface Electromyographic (EMG) activity was recorded using BioEMG equipment connected to laptop with installation of licensed BioPak analysis software (Digital Dental, Paddington, Australia) was used.

## Patient positioning:

Study subjects were seated in upright posture with Head Position (NHP) ensuring Frankfort horizontal plane parallel to the floor to standardize during the recordings.

## Skin preparation and electrode placement:

The skin surface at the electrode application site was prepared to reduce the impedance from oil secretions using 70% alcohol disinfectant. The electrode (ground) was placed on the muscle sternocleidomastoid. The other electrodes were spaced at a distance of 3 mm to 4 mm apart were made to adhere to the prepared surfaces, affixed bilaterally at the site to the most prominent muscle fibres temporalis, masseter and digastric muscles. The electrodes were positioned on the thickest part of muscle, aligned parallel to the muscle fibre direction. The placement site was verified through clinical palpation and functional assessment.

## Signal acquisition and amplification:

**Figure 1:**
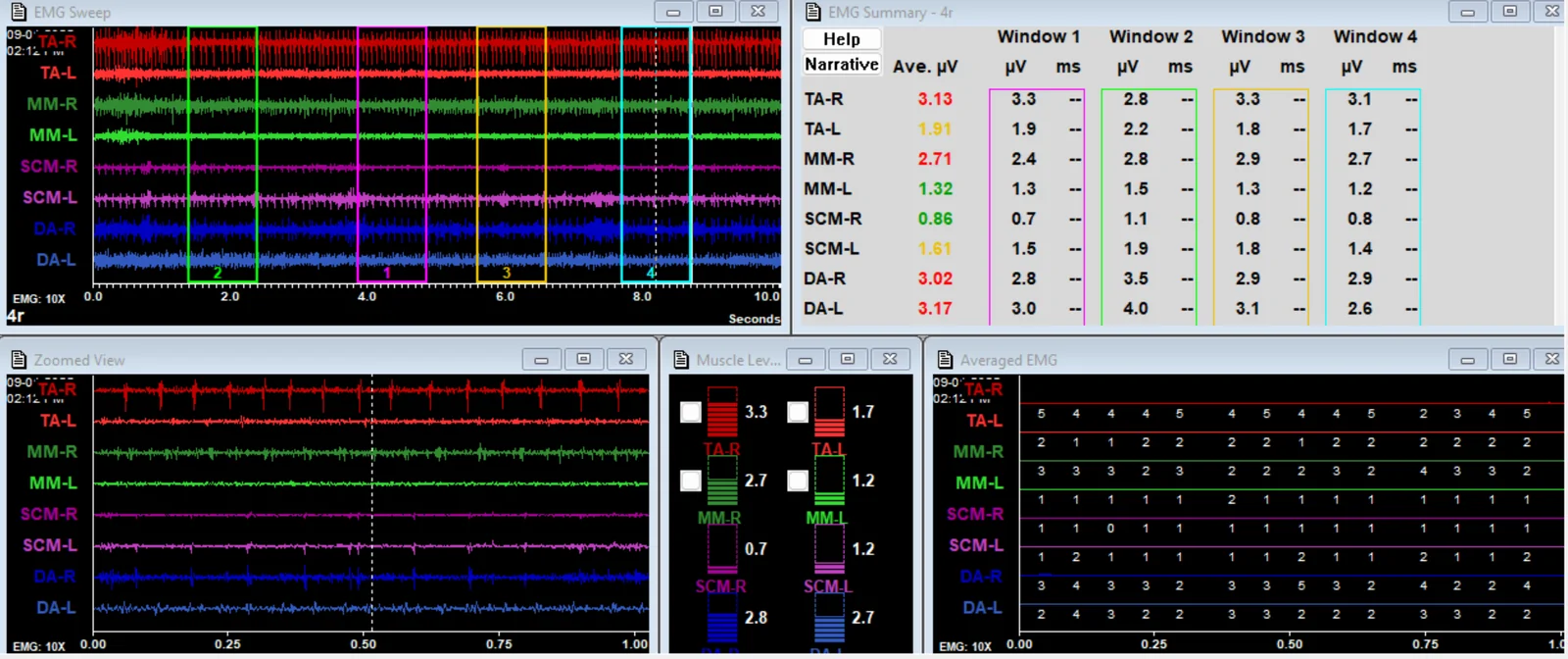
sEMG measurement Bio Pack software for REST or in CENTRIC RELATION

The electrode wires are inserted into circuit box through a microcontroller, attached to the laptop with installation of BioPak Software. With a fixed gain of 5,000, common-mode rejection ratio of hardware only > 130 dB at 60 Hertz, -3.0 to +3.0 volts DC common-mode voltage range and 30 to 1000 Hertz bandwidth (@ 2,000 Hz sample rate), signal-to-noise ratio of 1,000,000 to 1, differential amplifier was used to record signals. In order to reveal contraction pattern and relative intensities, the EMG software shows time domain windows as the signals and the average of three levels (Figure 1).

## Recording protocol:

At first, the muscle activity was recorded at rest, then during swallowing and teeth clenching, muscle activity was recorded. Both temporalis and masseter muscles was assessed for the simultaneous bilateral activity. The electrical activity at rest was measured using by the Resting EMG only, while the muscle activity during clenching and swallowing was measured in milliseconds. The contraction force was expressed as amplitude in microvolt units (Figure 1).

## Muscles assessed and duration of study:

The three muscles activity such as temporalis, masseter and digastric were assessed in centric relation (rest) at baseline (pre- treatment) and after 6 months of orthodontic treatment (post-treatment).

## Results and Discussion:

**Figure 2:**
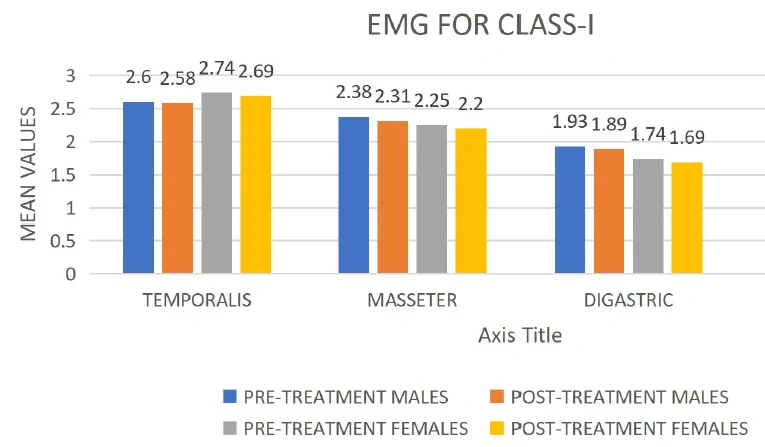
Pre and post treatment sEMG values for Temporalis, Masseter and Digastric in Class I male and female

**Figure 3:**
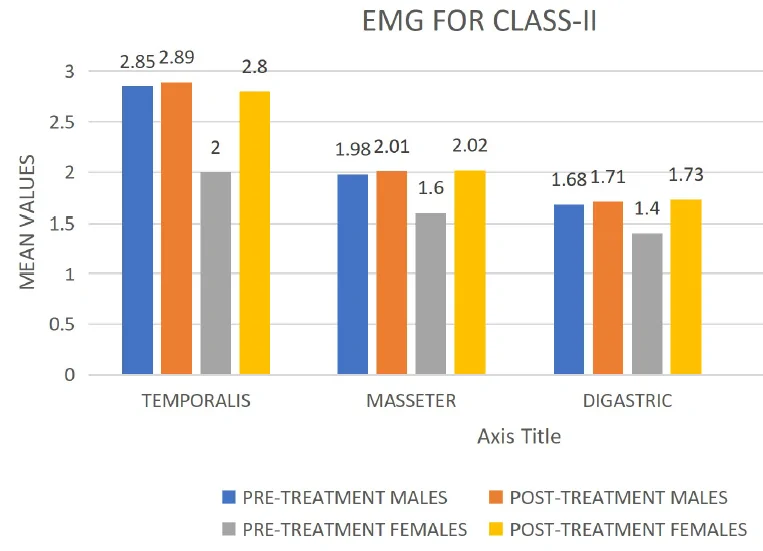
Pre and post treatment semg values for Temporalis, masseter and Digastric in Class II male and female

**Figure 4:**
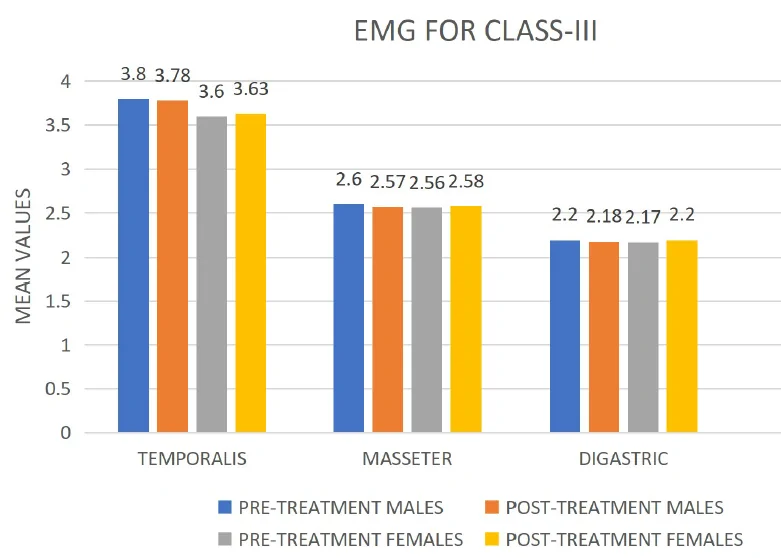
Pre and post treatment sEMG values for Temporalis, masseter and Digastric in Class III male and female

The normality of the data was assessed using Shapiro-Wilk test, it revealed normally distribution. Further analysis was done using parametric tests. To assess the overall differences between the groups paired t test was used. Electromyographic activity of the temporalis muscle in individuals treated with conventional braces (class I malocclusion) revealed no significant changes observed among the genders compared during pre- and post-treatment (Table 1). It shows the electromyography values for masseter muscle activity following orthodontic treatment with conventional braces among class I showed no significant changes seen in the group across both genders. Among the male subjects, the mean EMG activity showed a marginal reduction pre-treatment 2.38 ± 0.40 to post- treatment 2.31 ± 0.09 (t = -1.24, p = 0.32), while females subjects showed a comparable decrease from 2.25 ± 0.03 to 2.20 ± 0.10 (t = -1.14, p = 0.41). Although minimal reductions were observed, there was no statistical significance result which indicated conventional orthodontic therapy in class I did not influence the masseter muscle function suggested preservation of neuromuscular equilibrium following the treatment. On comparing the electromyography values for digastric muscle activity, including pre- and post-treatment conventional braces, among class I, no significant changes were seen between both the genders (Table 1 and Figure 2). Table 2 represents in Class II Study subjects, gender-specific variations were observed in the electromyographic activity following orthodontic treatment with conventional braces. For temporalis muscle activity, males subjects showed no significant differences between the pre-treatment (2.85 ± 1.1mm) and post- treatment values (2.89 ± 0.8mm) (p = 0.32), whereas females exhibited a significant increase in the activity from 2.00 ± 0.7mm to 2.80 ± 1.1mm (p = 0.01).Similarly, masseter muscle activity in males showed no significant changes (1.98 ± 1.3mm to 2.01 ± 0.2mm) (p = 0.28), while females subjects revealed an increase from 1.60 ± 0.8mm to 2.02 ± 0.4 mm (p = 0.05). For the digastric muscle, males presented a stable activity with no significant differences (1.68 ± 0.8mm to 1.71 ± 0.5 mm; t = 1.37, p = 0.63). Although, these findings indicates that neuromuscular adaptations following orthodontic therapy in Class II cases were more pronounced in females, particularly for the temporalis, masseter and digastric muscles suggesting a gender-related difference in functional response to treatment. (Table 2, Figure 3. In Table 3, the electromyographic (EMG) analysis for temporalis, masseter and digastric muscle activity in Class III subjects showed that there was no gender-specific change during treatment values. Although a slight decrease was observed in temporalis activity among males (3.80 ± 2.1mm to 3.78 ± 0.8mm) and increase in scores were observed in digastric activity among females (2.17 ± 1.1mm to 2.20 ± 0.5mm), these changes were not statistically significant. Overall, conventional orthodontic treatment did not significantly influence neuromuscular activity in Class III patients, suggesting for functional stability of masticatory muscles following the intervention (Table 3) (Figure 4).

By improving the dentofacial functions and esthetics, orthodontic treatment improves the quality of life of patients. Surface electromyography (EMG) is a reliable, non-invasive method used for evaluating muscle activity by detecting the electrical potential through electrodes placed on the skin. Surface EMG has gained popularity in dentistry due to the simplicity of the procedure [6]. This technique measured the magnitude and duration of muscle activity by recording the intrinsic electrical potential from the active motor units [5]. Orthodontic leveling and alignment influence the activity of muscles, demonstrating a dynamic neuromuscular adaptation during treatment [17]. In Angles Class I and Class III malocclusions revealed no significant changes observed in muscle activity in pre- and post-treatment recordings, which suggested that orthodontic therapy in these groups largely preserves neuromuscular equilibrium [7]. Previous studies reported these findings are consistent with stability of masticatory muscle function in Class I and III after orthodontic corrections, where occlusal changes may be less demanding on neuromuscular adaptation. Class II patients, however showed a statistically significant difference in sEMG of temporalis pre and post treated individuals with an increased post treatment value, this is similar to previous study which concluded that the Temporalis muscle activity of Class II malocclusion patient was markedly increased in orthodontically treated patients than non-treated patients [8]. Class II patients showed a significant change in sEMG of masseter muscle in pre and post treated individuals with an increased post treatment value. These results were similar to the study conducted by Ocak *et al.* which states that left and right masseter activity decreases in Class II/div1 after protraction and increases after the treatment to average range [9]. The results were also partially similar to the study conducted by Buxbaum *et al.* [8] suggested the average amplitude level for anterior temporalis muscles and the masseter muscle was 12.9mV and 10-8mV. Class II patients showed a significant change in sEMG of digastric muscles in pre and post treated individuals with an increased post treatment value. Similarly, a previous study revealed the digastric muscle was found to be greater in the Class II group [10]. Class II patients showed a significant difference in sEMG of three muscles (temporalis, masseter and digastric) pre and post treated individuals [11]. It was also observed that as the treatment progresses in Class II there was a gradual increase in muscle activity [12]. Previous studies suggest that there was a correlation between centric slide and muscle activity also states that these are inversely proportional to each other [13]. Based on the above reference, this study shows the significant correlation between centric slide and muscle activity [14]. It was observed that the centric slide gradually decreased 6 months into orthodontic treatment on the other hand there was significant increase in muscle activity thus indicating the inverse proportionality [15].

## Conclusion:

We show that surface electromyography is a reliable tool to evaluate the neuromuscular responses before and after orthodontic treatment. These findings revealed that Class I and Class III malocclusion subjects maintained a stable EMG activity following conventional orthodontic therapy. It suggested a preservation of neuromuscular equilibrium. Conversely, Class II subjects particularly females exhibited significant increases in temporalis, masseter and digastric muscle activity post-treatment indicating a gender-related functional adaptation. Future studies to explore their clinical relevance for individualized orthodontic treatment planning.

## Clinical significance:

Understanding how activity as muscle measured by surface electromyography (sEMG) changes before and after orthodontic treatment is not merely academic: it has direct bearing on patient function, comfort and long-term outcomes. By quantifying shifts in neuromuscular balance associated with occlusal changes, this study can illuminate whether the orthodontic correction restores harmonious muscle activation or inadvertently introduces new imbalances. The insights may guide clinicians in refining force application, customizing finishing protocols, and optimizing retention strategies in a way that aligns not only with structural alignment but also with functional stability and patient well-being.

## Figures and Tables

**Table 1:** Shows the electromyography values for 3 muscle activity, including pre- and post-treatment conventional braces among class I between both genders using paired t test.

**EMG values for 3 muscle activity (Class-I)**						
**EMG for muscle assessed**	**Gender**	**N**	**Pre treatment Mean ± SD**	**Post treatment Mean ± SD**	**t value**	**p value**
EMG for Temporalis muscle (Class-I)	Male	5	2.60 ± 0.2	2.58 ± 0.5	1.78	0.68
	Female	5	2.74 ± 0.1	2.69 ± 0.09	2.2	0.38
EMG for Masseter muscle (Class-I)	Male	5	2.38 ± 0.4	2.31 ± 0.09	-1.24	0.32
	Female	5	2.25 ± 0.03	2.20 ± 0.1	-1.14	0.41
EMG for Digastric muscle (Class-I)	Male	5	1.93 ± 0.5	1.89 ± 0.1	-0.76	0.68
	Female	5	1.74 ± 0.1	1.69 ± 0.08	0.68	0.53
*Significant level p < 0.05.

**Table 2:** Shows the electromyography values for 3 muscle activity, including pre- and post-treatment conventional braces among class II between both genders using paired t test

**EMG values for 3 muscle activity (Class-II)**						
**EMG for muscle assessed**	**Gender**	**N**	**Pre treatment Mean ± SD**	**Post treatment Mean ± SD**	**t value**	**p value**
EMG for Temporalis muscle (Class-II)	Male	5	2.85 ± 1.1	2.89 ± 0.8	2.05	0.32
	Female	5	2.00 ± 0.7	2.80 ± 1.1	11.5	0.01*
EMG for Masseter muscle (Class-II)	Male	5	1.98 ± 1.3	2.01 ± 0.2	3.08	0.28
	Female	5	1.60 ± 0.8	2.02 ± 0.4	12.74	0.05*
EMG for Digastric muscle (Class-II)	Male	5	1.68 ± 0.8	1.71 ± 0.5	1.37	0.63
	Female	5	1.40 ± 0.5	1.73 ± 0.3	16.02	0.00*
*Significant level p < 0.05

**Table 3:** Shows the electromyography values for temporalis muscle activity, including pre- and post-treatment conventional braces among class III between both genders using paired t test.

**EMG values for 3 muscle activity (Class-III)**						
**EMG for muscle assessed**	**Gender**	**N**	**Pre treatment Mean ± SD**	**Post treatment Mean ± SD**	**t value**	**p value**
EMG for Temporalis muscle (Class-III)	Male	5	3.80 ± 2.1	3.78 ± 0.8	2.12	0.16
	Female	5	3.60 ± 1.4	3.63 ± 1.3	4.91	0.28
EMG for Masseter muscle (Class-III)	Male	5	2.60 ± 0.5	2.57 ± 1.3	3.69	0.87
	Female	5	2.56 ± 1.1	2.58 ± 1.2	2.01	0.42
EMG for Digastric muscle (Class-III)	Male	5	2.20 ± 0.4	2.18 ± 0.8	4.9	0.17
	Female	5	2.17 ± 1.1	2.20 ± 0.5	6.87	0.32
